# BMC Immunology reviewer acknowledgement, 2015

**DOI:** 10.1186/s12865-016-0140-5

**Published:** 2016-02-03

**Authors:** Guangde Tu

**Affiliations:** Room 1504-1505 Financial Plaza, No. 333 Jiujiang Road, Huangpu District Shanghai, 200001 China

## Abstract

The editors of BMC Immunology would like to thank all our reviewers who have contributed their time to the journal in Volume 16 (2015).

**Sunil K. Ahuja**

USA

**Irving Allen**

USA

**Safinur Atay**

USA

**Robert Axtell**

USA

**Agnes Azimzadeh**

USA

**Najib Aziz**

USA

**Dolgor Baatar**

USA

**Karl Balabanian**

France

**Raghavendran Balaji**

Malaysia

**Maria Vittoria Barone**

Italy

**Kanthesh Basalingappa**

India

**Vira Bitko**

USA

**Gwenoline Borhis**

France

**Belaid Bouazza**

USA

**Todd Brusko**

USA

**Maria Bucova**

Slovakia

**Luigi Buonaguro**

Italy

**Chuanhai Cao**

USA

**Ennio Carbone**

Italy

**Stefano Caserta**

United Kingdom

**Flora Castellino**

USA

**Jan Ceuppens**

Belgium

**Lung-Ji Chang**

USA

**Karlhans Fru Che**

Sweden

**Gerald Kimani Chege**

South Africa

**Huaiyong Chen**

China

**Remi Cheynier**

France

**Eun Young Choi**

Republic of Korea

**Elisabeth Cramer Bordé**

France

**Julio Croda**

Brazil

**Weiguo Cui**

USA

**Pradeep Dagur**

USA

**Silvia Daher**

Brazil

**Volker Daniel**

Germany

**Randall Davis**

USA

**Cyro de Brito**

Brazil

**Deepak Deshpande**

USA

**Alvaro Diaz**

Uruguay

**Paul Drain**

USA

**Caroline Dunk**

Canada

**Shannon Dunn**

Canada

**Ravi Dyavar Shetty**

USA

**Hossam Ebaid**

Saudi Arabia

**Ganapathy Ekambaram**

USA

**Shankar Esaki Muthu**

Malaysia

**Mohamed Ezzelarab**

USA

**Joshua Fierer**

USA

**Greg Finak**

USA

**Graziano Fiorito**

Italy

**Patrick Forde**

Ireland

**Martyn French**

Australia

**Jorge Galindo-Villegas**

Spain

**Arunakumar Gangaplara**

USA

**Olivier Garraud**

France

**Frank Geller**

Denmark

**Biswarup Ghosh**

Philippines

**Andrea Gioffredi**

Italy

**Ricardo Gomez**

Argentina

**Richard Goodman**

USA

**Cécile Gouttefangeas**

Germany

**Hasan Tahsin**

Turkey

**Markus Graeler**

Germany

**Christina Graves**

USA

**Neil Greenspan**

USA

**Alisa Gruden-Movsesijan**

Serbia

**Gerald Gruetz**

Germany

**Julius Hafalla**

United Kingdom

**Bruce Hall**

Australia

**Laurie Harrington**

USA

**Catherine Hawrylowicz**

United Kingdom

**Zhiheng He**

USA

**Amy Hobeika**

USA

**Peter Hoet**

Belgium

**Markus Hoffmann**

Germany

**Jessica Howell**

Australia

**Shao-Chung Hsia**

USA

**Jeremy Hughes**

United Kingdom

**Zsolt Illes**

Denmark

**David J. Klinke**

USA

**Joseph Jarvis**

South Africa

**Richard Jaspers**

Netherlands

**Ki Jun Jeong**

Republic of Korea

**Narayanaperumal Jeyaparthasarathy**

India

**Jim Kaufman**

United Kingdom

**Marcus Kaul**

USA

**Tadamitsu Kishimoto**

Japan

**David Kluth**

United Kingdom

**Dietmar Knopp**

Germany

**Vjollca Konjufca**

USA

**Peter Korosec**

Slovenia

**Rami Kridli**

Canada

**Atsushi Kumanogoh**

Japan

**Alan Lazarus**

Canada

**Terence Lee**

Hong Kong, China

**Eun-Hyung Lee**

USA

**Bernd Lepenies**

Germany

**James Li**

Hong Kong, China

**Bing Li**

USA

**Lalita Limaye**

India

**Siqi Liu**

USA

**Yuanhua Liu**

China

**Fu-tong Liu**

USA

**Lorenzo Lorusso**

Italy

**Andreas Lundqvist**

Sweden

**Adrian Luty**

France

**Hadi Maazi**

USA

**Paul MacNorman**

USA

**Holden Maecker**

USA

**Rizgar Mageed**

United Kingdom

**Ravishankar Ram Mani**

Malaysia

**Palanikumar Manoharan**

USA

**Nicholas Mantis**

USA

**David Margulies**

USA

**Per Marits**

Sweden

**Olivia Martinez**

USA

**Patricia Martins**

Brazil

**Chandirasegaran Massilamany**

USA

**Nivedita Menon**

Malaysia

**Jim Middleton**

United Kingdom

**Leticia Moreno-Fierros**

Mexico

**Gerwyn Morris**

United Kingdom

**Adrian Mountford**

United Kingdom

**Satoru Nagatoishi**

Japan

**Subhadra Nandakumar**

USA

**Norman Nausch**

Germany

**Nanda Kumar**

USA

**Maria Navarro**

Spain

**Akio Niimi**

Japan

**Tatsuji Nishihara**

Japan

**David Nolan**

Australia

**Elio Novembre**

Italy

**Kenneth Oestreich**

USA

**Shinji Oki**

Japan

**Taishi Onodera**

Japan

**Enrique Ortega**

Mexico

**Ferda Ozkinay**

Turkey

**Tammy Ozment**

USA

**Sudheer Kumar Pabbisetty**

USA

**Senthilkumar Palaniyandi**

USA

**Kirstin Parkin**

USA

**C. David Pauza**

USA

**Eric Peterson**

USA

**Deborah Phippard**

USA

**Vanessa Pirrone**

USA

**Bruno Pozzetto**

France

**Immo Prinz**

Germany

**Michal Rahat**

Israel

**Chander Raman**

USA

**Giuseppe Remuzzi**

Italy

**Joseph Reynolds**

USA

**Kirsi Rilla**

Finland

**Lijun Rong**

USA

**Stephan Rosshart**

USA

**Cansin Sackesen**

Turkey

**Hirohisa Saito**

Japan

**Juan Salazar**

USA

**Chandramathi Samudi**

Malaysia

**Philipp Sand**

Germany

**Saikolappan Sankaralingam**

USA

**Alexandra Santos**

United Kingdom

**Douaa Sayed**

Egypt

**Anja Scholzen**

Netherlands

**Gesmar Segundo**

Brazil

**Patrícia Severino**

Brazil

**Ho Sham**

USA

**Zahra Sharifzadeh**

Iran

**Prashant Sharma**

India

**David Sherr**

USA

**Peter Silverstein**

USA

**Udai Singh**

USA

**Nagendra Singh**

USA

**Krzysztof Sladek**

Poland

**Joyce Solheim**

USA

**Pejman Soroosh**

USA

**Boryana Stamova**

USA

**Jennifer Stow**

Australia

**Zuoming Sun**

USA

**Hong Yien Tan**

Malaysia

**Ichiro Taniuchi**

Japan

**Robert Thimme**

Germany

**Kanyarat Thueng-in**

Thailand

**Omar Tliba**

USA

**Piotr Trzonkowski**

Poland

**Homer Twigg III**

USA

**Raivo Uibo**

Estonia

**Shigeki Umemura**

Japan

**Eva Untersmayr**

Austria

**Dana Vanlandingham**

USA

**Dewton Vasconcelos**

Brazil

**Vijayakumar Velu**

USA

**Saguna Verma**

USA

**Miguel Vicente-Manzanares**

Spain

**Joshua Vieth**

USA

**Ramachandran Vignesh**

India

**Armando Villalta**

USA

**Aristo Vojdani**

USA

**Mark Wallet**

USA

**Ji-Yang Wang**

China

**Shifeng Wang**

USA

**Ola Winqvist**

Sweden

**Linda Wittkop**

France

**Won Fen Wong**

Malaysia

**James Wynne**

Australia

**Zhou Xing**

Canada

**Gur Yaari**

USA

**Chul Woo Yang**

Republic of Korea

**Pingchang Yang**

China

**Yong Yean Kong**

Malaysia

**Weiming Yuan**

USA

**Xingxing Zang**

USA

**Margit M. Zeher**

Hungary

**Chun Zeng**

USA

**Youming Zhang**

United Kingdom

**Xuyu Zhou**

China

**Jochen Zwerina**

Austria

